# Pruritus in Uremic Patients: Approaches to Alleviating a Common Symptom in Chronic Kidney Disease

**DOI:** 10.3390/life15071001

**Published:** 2025-06-24

**Authors:** Ștefania Cîrstea, Olguța Anca Orzan, Diana Silvia Zilișteanu

**Affiliations:** 1 Faculty of Medicine, ‘Carol Davila’ University of Medicine and Pharmacy, 020021 Bucharest, Romania; stefania.cirstea@rez.umfcd.ro (Ș.C.); diana.zilisteanu@umfcd.ro (D.S.Z.); 2 Department of Allergology, Nicolae Malaxa Clinical Hospital, 022441 Bucharest, Romania; 3 Department of Dermatology, ‘Elias’ University Emergency Hospital, 011461 Bucharest, Romania; 4 Nephrology Department, Fundeni Clinical Institute, 022328 Bucharest, Romania

**Keywords:** pruritus, chronic kidney disease, uremic patient, uremic pruritus

## Abstract

Chronic kidney disease-associated pruritus (CKD-aP) is a distressing symptom that affects both dialysis and non-dialysis patients, significantly impairing their quality of life. Despite its multifactorial pathophysiology, no gold-standard treatment has been established. This review explores various therapeutic options and evaluates their effectiveness based on recent clinical studies and meta-analyses. Therapies targeting novel mechanisms have evolved in recent years. Difelikefalin, a κ-opioid receptor agonist, represents a breakthrough in systemic treatment, demonstrating efficacy with a favorable safety profile. Another opioid-based therapy, nalfurafine, has shown notable symptom relief in multiple clinical studies, with a low risk of abuse. Sertraline, an antidepressant, offers another alternative, although its delayed onset remains a limitation. Nonpharmacologic approaches are also evolving. Phototherapy, particularly UV-B therapy, modulates the immune response, reduces inflammation, and effectively alleviates itching in hemodialysis patients. Personalized treatment strategies are crucial, as responses vary among patients. Further research, including comparative and long-term studies, is essential to refine treatment algorithms and improve patient outcomes. By integrating new pharmacologic and nonpharmacologic options, CKD-aP management is shifting toward a more tailored and effective approach that addresses the individual needs of each patient.

## 1. Introduction

Chronic kidney disease (CKD) affects over 10% of the global population—more than 800 million people [1]. It is more common in the elderly, women, racial minorities, and individuals with arterial hypertension or diabetes [1], posing a significant burden on public health systems, especially in low- and middle-income countries.

Pruritus is one of the most distressing symptoms in CKD patients, particularly in those undergoing dialysis, affecting around 60% [2]. Known as uremic pruritus or CKD-associated pruritus (CKD-aP), it severely impacts quality of life, contributing to sleep disturbances, depression, and even increased mortality [2].

The pathophysiology of CKD-aP is not fully understood, with proposed mechanisms including dermatological, inflammatory, neurological, metabolic, and electrolyte-related factors. As no gold standard treatment exists, and responses vary widely, management remains challenging. Still, progress has been made, and a range of topical, systemic, and non-pharmacologic options are available.

Despite this, CKD-aP remains under-researched, with a need for more comparative and long-term studies. Individualized treatment plans are crucial for addressing the diverse responses among patients.

## 2. Materials and Methods

This review explores various therapeutic options and evaluates their effectiveness based on recent clinical studies and meta-analyses. It is organized as follows. It first examines each pathophysiological mechanism, followed by a discussion of the corresponding therapeutic interventions targeting these mechanisms (Table 1). We conducted extensive research within major databases (PubMed, Google Scholar, and ResearchGate) using the keywords ‘uremic pruritus treatment,’ ‘pruritus kidney disease,’ ‘uremic pruritus,’ ‘pathophysiology pruritus kidney disease,’ ‘difelikefalin in pruritus,’ ‘pregabalin uremic pruritus,’ ‘sertraline in pruritus,’ and ‘nalfurafine in pruritus.’ Only studies published after 2020 were included in this analysis. We primarily focused on the potential therapies that have been increasingly incorporated into the latest clinical trials and have demonstrated promising outcomes with high efficacy. This review did not include a comprehensive analysis of older therapies that yielded uncertain results or were supported by low-confidence evidence.

## 3. Pathological Mechanisms in CKDaP

In the pathogenesis of CKDaP, it is suggested that the normal balance may be disrupted by multiple factors [3,4,5,6], including the following:

1. dermatological (skin barrier dysfunction and xerosis);
2. uremic toxins and metabolite accumulation;
3. inflammatory (increased pro-inflammatory cytokines, immune system abnormality);
4. neurological (μ-opioid overexpression and κ-opioid downregulation, neuropathy).

## 4. Skin Abnormalities in Patients with End-Stage Kidney Disease (ESKD)

Chronic renal failure and dialysis affect the skin and its appendages (hair, nails, and secretory glands), leading to xerosis, ecchymoses, pigmentary abnormalities, fragile hair and nails, and delayed wound healing, reflecting impaired tissue perfusion. In addition, microangiopathic changes, such as basement membrane thickening and endothelial damage in capillaries, arterioles, and venules are observed across all stages of CKD and tend to worsen over time with dialysis.

Xerosis arises due to secretory gland atrophy, elevated skin pH, and decreased hydration of the stratum corneum, although the skin’s barrier function typically remains intact. Pathological remodeling of cutaneous capillaries is likely to be driven by systemic factors such as endocrine disturbances (e.g., calcium–phosphate imbalance and elevated parathyroid hormone), immune dysfunction, hypertension, and accumulated metabolites. This microangiopathy leads to capillary rarefaction and fibrosis in the deeper dermis, further aggravating skin dryness and contributing to the progression of xerosis [3].

Recent studies have investigated whether daily skincare with moisturizers and rehydrating creams improves uremic pruritus.

**Figure 1:**
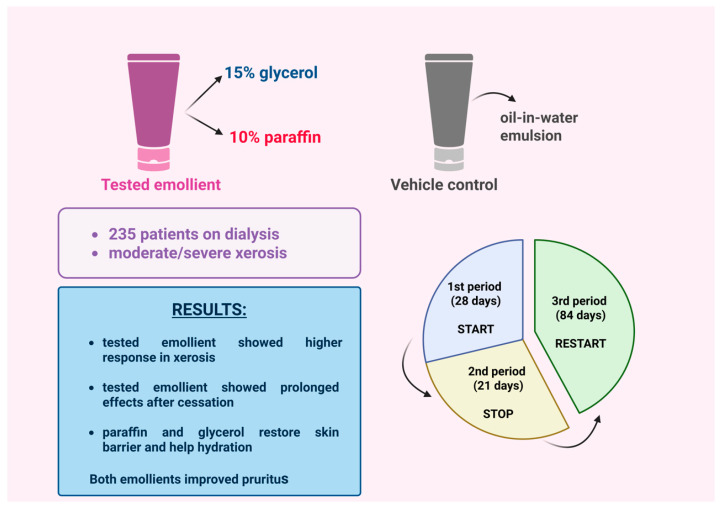
A study investigated the long-term efficacy of an emollient containing glycerol and paraffin for moderate-to-severe uremic xerosis [7]. (Figure created in https://BioRender.com).

A phase 3, randomized, double-blind, vehicle-controlled study conducted across multiple sites in Europe investigated the efficacy of an emollient (V0034CR) containing 15% glycerol and 10% paraffin in the long-term alleviation of uremic xerosis and uremic pruritus [7]. The study had three phases and included 235 dialysis patients with moderate to severe xerosis (Figure 1).

In Phase 1 (28 days), patients applied either V0034CR or a control emulsion once daily. The V0034CR group showed a higher response rate (60% vs. 41%) and greater improvement in xerosis [7].

Phase 2 (21-day break) revealed some lasting effects in the V0034CR group, but symptoms relapsed, with no significant difference between groups—suggesting partial restoration of the skin barrier.

In Phase 3 (84 days), reapplication of V0034CR restored the initial benefits. Pruritus decreased significantly in both groups, probably due to the emulsion base, and quality-of-life scores improved. These results support V0034CR as a promising option for uremic xerosis and pruritus [7].

**Figure 2:**
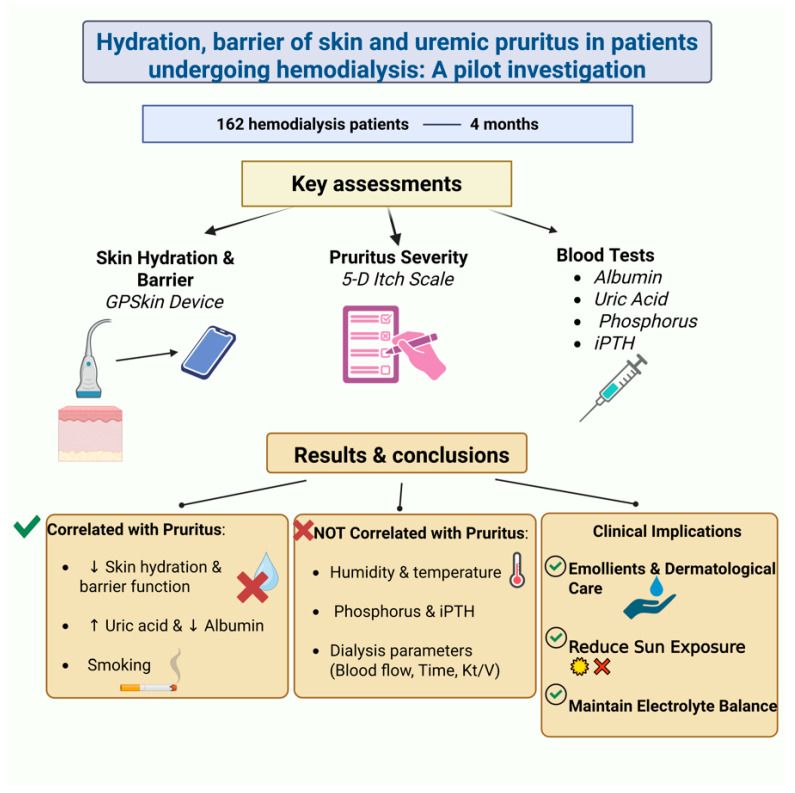
A cross-sectional study explored the relationship between skin barrier function, hydration, and pruritus in CKD patients undergoing hemodialysis [8]. *↑, Increase; ↓, decrease.* (Figure created in https://BioRender.com).

A cross-sectional study in Taiwan investigated the relationship between skin barrier function, hydration, and pruritus in 162 hemodialysis patients over four months [8]. Using a GPSkin device, researchers measured stratum corneum hydration (SCH) and transepidermal water loss (TEWL), while patients also completed lab tests and the 5-D itch scale to assess their pruritus (Figure 2).

That study found a strong link between impaired skin barrier function, low hydration, and increased pruritus in hemodialysis patients, and no correlation with environmental or dialysis-related factors. While phosphorus and iPTH levels showed no association with itching, pruritus correlated significantly with uric acid and albumin levels. Smoking and uric acid were key predictors of itch severity. The authors highlighted the importance of skin hydration and barrier integrity in managing CKD-aP and recommended regular skincare, emollient use, and dermatological follow-up.

## 5. Metabolic Abnormalities in Patients with CKD

Recent research has shown a correlation between impairment of kidney detoxification functioning and the development of itching. In some studies, the increase of dialysis dose measured by Kt/V correlated with lesser intensity of pruritus, which means that filtration of certain metabolites (e.g., PTH, β2-microglobulin [4,9]) could help reduce the intensity of pruritus [3,10]. An increase in PTH serum levels due to calcium–phosphate imbalance in CKD may be responsible for dermal calcium deposition and facilitate development of pruritus [3]. Zinc was thought to be another possible culprit, but it was later proven that its decrease was not correlated with pruritus [3,11]. It has been observed that exposure to the acidotic environment in CKD through specific proton receptors can lead to triggering and intensification of pruritus [3]. Nevertheless, iron deficiency and anemia can trigger increases in IL-6 and hepcidin levels, which can resemble chronic inflammation status [3,12,13].

Recent studies increasingly indicate that serum levels of various blood components have a limited effect on the intensity of pruritus. Several clinical trials assessing the efficacy and safety of different therapies for uremic pruritus have included blood parameter measurements in their protocols, and their findings are quite striking, as discussed in this review.

One study noted that low albumin and high uric acid levels were significant factors influencing the intensity of pruritus, while no statistical correlation was observed between pruritus and serum levels of phosphorus, PTH, or dialysis parameters such as total dialysis time, blood flow rate, or clearance efficiency (Kt/V) [8].

Another study found a positive correlation between itch intensity and serum levels of urea and ferritin but no correlation between pruritus and other blood values (calcium, phosphorus, PTH, serum albumin, and Kt/V). The authors attribute this finding to patient selection criteria [14].

As observed, additional investigation is necessary to establish a definitive conclusion about the relationship between blood components, dialysis parameters, and the occurrence of pruritus.

## 6. Immune System Abnormalities in ESKD

Acute pruritus is primarily histamine-driven, caused by histamine release onto sensory neuron receptors. Histamine, secreted by mast cells, is a well-known pruritic mediator, especially in conditions like urticaria, where it causes itching, swelling, and rash. In contrast, CKD-associated pruritus (CKDaP) typically presents with itching alone [3,4]. Clinical trials have shown that antihistamines are largely ineffective for treating CKDaP [4]. Although mast cells are elevated in the serum and dermis of CKD patients, this does not correlate with the severity of pruritus [3]. The limited efficacy of antihistamines and lack of association between histamine markers and itch intensity highlight the need to investigate alternative mechanisms in CKDaP pathophysiology [6].

Chronic pruritus is linked to a persistent inflammatory state that promotes the release of various immunomodulatory factors. Among these, interleukin-31 (IL-31) has received particular attention. Produced by CD4+ TH2 cells and mast cells, IL-31 activates IL-31Rα and oncostatin M receptor β on sensory neurons, triggering itching. This activation also stimulates the release of neuropeptides like substance P, which further activates mast cells. Additionally, cytokines such as IL-4 and IL-13 increase neuronal sensitivity to pruritic stimuli, amplifying the itch response. These self-sustaining cycles of interleukins, neuropeptides, and proteases contribute to the chronic and debilitating nature of pruritus [6].

Mast cell activation contributes to a persistent state of microinflammation, leading to the release of neuropeptides and tachykinins, such as substance P, which further stimulate the transcription and translation of proinflammatory cytokines like IL-2 [3]. Other contributors to microinflammation include protein processing abnormalities, oxidative stress, and chemical imbalances caused by dialysis [3].

In CKD, an increased Th1/Th2 lymphocyte ratio and elevated levels of inflammatory markers have been reported, including C-reactive protein (CRP), IFN-γ, IL-2, IL-6, and tumor necrosis factor-α (TNF-α), which are secreted by Th1 lymphocytes. Additionally, increased IL-31 levels secreted by Th2 lymphocytes have been observed [3,4].

Studies have shown that nemolizumab, an anti-IL-31 antibody, reduces sensation of pruritus [15]. Conversely, subcutaneous administration of IL-2 has been found to induce itching [4,16]. Based on these findings, it has been speculated that multiple inflammatory cytokines, including IL-2, IL-4, IL-6, IL-13, and IL-31, may play a role in the pathogenesis of pruritus. Furthermore, elevated inflammatory markers such as Th1 cells, CRP, leukocytosis, ferritin, and decreased albumin levels have been associated with this condition [3,4]. Therefore, pruritus may be a direct consequence of these microinflammatory changes.

The inflammatory hypothesis emerged after observing that certain immunosuppressive therapies targeting immune pathways—such as ultraviolet therapy and tacrolimus—effectively reduced pruritus intensity and prevalence [3,17]. A similar effect was noted in patients with glomerulopathies who received immunosuppressive medication to reduce inflammation [3,18].

Ultraviolet B (UVB) therapy has been shown to exert an immunomodulatory effect by reducing proinflammatory cytokines, inhibiting Th1 and Th2 cell activity, and inducing mast cell apoptosis [2,19].

**Figure 3:**
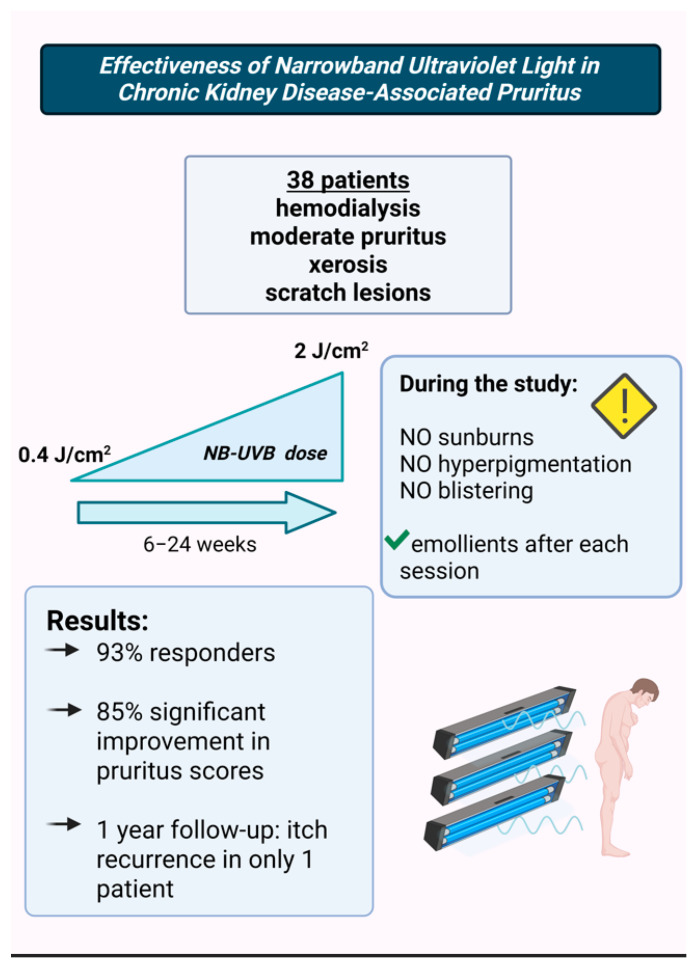
The study that assessed the efficacy of NB-UVB therapy in CKD-aP [20]. (Figure created in https://BioRender.com).

A 2024 study on 38 CKD patients undergoing hemodialysis, who also had moderate pruritus, xerosis, and scratch lesions, evaluated the effects of biweekly narrowband ultraviolet B (NB-UVB) phototherapy. Improvements in itching were observed starting from the sixth session [20] (Figure 3). Patients received an initial dose of 0.4 J/cm^2^, which was gradually increased to a maximum of 2 J/cm^2^, depending on their erythema response.

The study protocol carefully aimed to prevent adverse effects such as sunburn, hyperpigmentation, and blistering, and patients were advised to apply emollients after each session. The average number of sessions was 13 (ranging from 6 to 24). By the end of the study, 93% of patients were classified as responders, with 85% showing significant improvement in the intensity, frequency, and extent of pruritus, as well as better sleep quality. At the 1-year follow-up, only one out of 38 patients experienced pruritus recurrence.

These findings align with previous studies emphasizing the efficacy of phototherapy as a treatment for CKD-aP.

**Figure 4:**
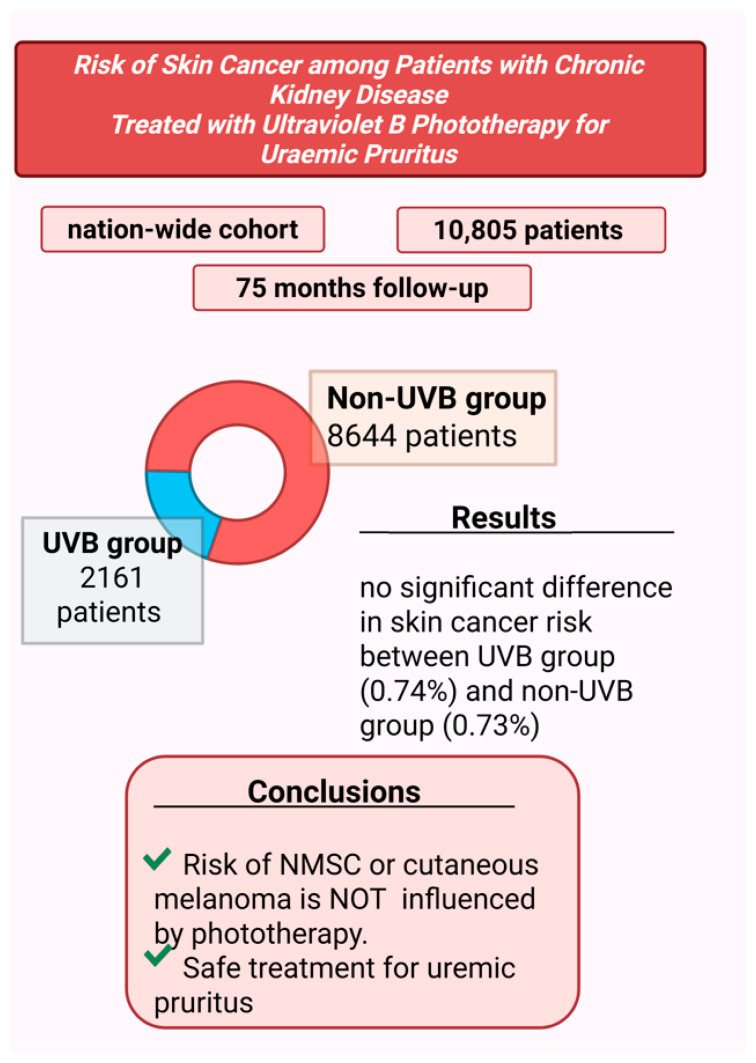
Assessment of risk of cutaneous cancer in patients receiving phototherapy for pruritus treatment [21] (Figure created in https://BioRender.com).

However, concerns about the potential carcinogenic effects of UVB phototherapy remain. A nationwide cohort study of 10,805 patients with CKD-aP, followed for a median period of 75 months, assessed the carcinogenic risk associated with UVB therapy [21], (Figure 4). The results showed no significant correlation between UVB phototherapy and increased risk of skin cancer, including both non-melanoma skin cancer (NMSC) and cutaneous melanoma.

That study divided patients into two groups: 2161 in the UVB-treated group and 8644 in the non-UVB group. During the follow-up period, skin cancer was diagnosed in 0.74% of patients in the UVB group and 0.73% in the non-UVB group, indicating no statistically significant difference.

The authors highlighted several strengths of their study, including the large sample size, extended follow-up duration, integration of cancer and death registry data for more accurate outcome assessment, and the use of rigorous statistical methods. Based on these findings, the study concluded that UVB phototherapy did not increase the risk of NMSC or melanoma, supporting its safety as a treatment option for uremic pruritus.

## 7. Sensory Pathways and Neurotransmitters in Uremic Pruritus and Pain Transmission

Sensory neurons are activated by the above-mentioned pruritogens and are divided into two categories: histamine-dependent and histamine-independent [4,22]. This distinction helps explain the lack of response to antihistamine treatment in patients with uremic pruritus.

Histamine-independent impulses follow the same transmission pathway as nociceptive stimuli, traveling through polymodal unmyelinated C fibers. These fibers relay the impulse to secondary sensory neurons in the dorsal ganglia, leading to the release of neurotransmitters in the synaptic cleft, including glutamate, substance P, gastrin-releasing peptide, and B-type natriuretic polypeptide [4].

Serotonin can further stimulate receptors in the spinothalamic tract, perpetuating itch mechanisms. Elevated serotonin levels have been found in CKDaP [3], leading some publications to propose serotonin as a possible contributor to pruritus [4,23]. However, data on the efficacy of the serotonin receptor antagonist ondansetron in controlling itching remains contradictory [3,4,24]. Some patients with various types of pruritus (uremic, opioid-induced, and cholestatic) have reported relief of symptoms with 5-HT3 antagonists, but evidence supporting their effectiveness is still limited [6].

### 7.1. Opioid System Dysregulation

Both central and peripheral nerves as well as several skin cells (melanocytes, keratinocytes, peripheral mast cells) contain opioid receptors, which play a key role in the development of pruritus. As a result, these receptors have gained increasing attention as potential therapeutic targets in recent years [3].

While discussing opioid receptors and their effects on the brain may seem excessive in this context, it is essential for a comprehensive understanding of how certain central nervous system-acting drugs alleviate pruritus in CKD patients, as well as the potential adverse effects associated with these treatments.

**Figure 5:**
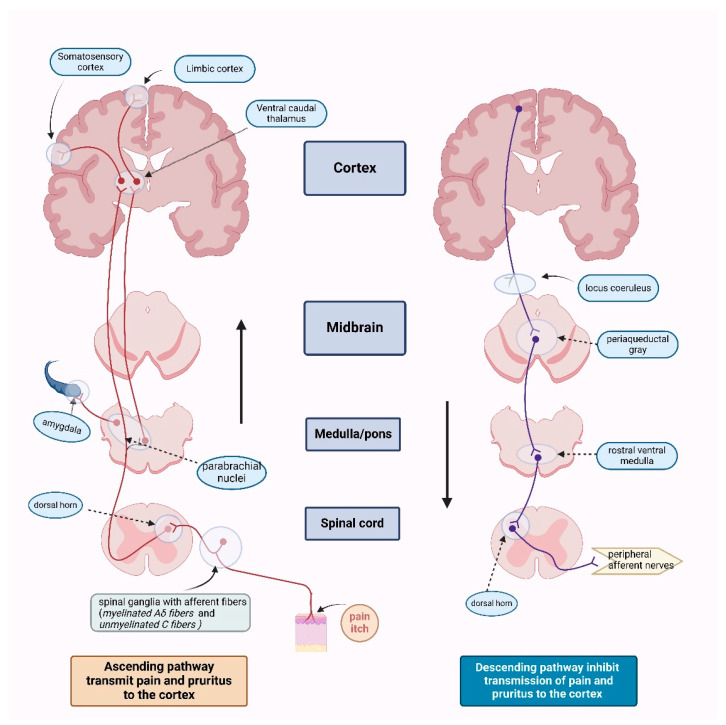
Ascending and descending pathways for both pain and pruritus [27] (Figure created in https://BioRender.com).

Pain stimuli are transmitted from the periphery by afferent neurons located in the spinal ganglia, traveling through specific fibers: myelinated Aδ fibers for sharp, localized pain and unmyelinated C fibers for diffuse, poorly localized pain [25,26,27] (Figure 5). The ascending pathways, formed by axons of neurons in the dorsal horn of the spinal cord, synapse with third-order neurons in the ventral caudal nucleus of the thalamus or with parabrachial nuclei in the medulla or pons [27]. From there, different thalamic regions project to the somatosensory cortex and limbic system, where pain stimuli are processed into conscious sensations.

At each synaptic level of the pain transmission pathway, descending inhibition modulates the signal, reducing pain perception [27]. This mechanism involves opioid receptors located in various structures of the brainstem (periaqueductal gray, locus coeruleus, and rostral ventral medulla), the dorsal horn of the spinal cord (specifically the substantia gelatinosa), and throughout the peripheral afferent nerves [26].

Opioid receptors are classified into three main pharmacological categories: μ (mu) receptors (MOR), δ (delta) receptors (DOR), and κ (kappa) receptors (KOR). These receptors contribute to antinociception at both spinal and supraspinal levels. They are distributed along the ascending pathways (primary afferents and spinal cord pain transmission neurons) and descending pathways (neurons in the midbrain and medulla), which modulate pain perception [26,27]. Additionally, opioid receptors influence pain reactivity via their presence in the basal ganglia, hypothalamus, limbic system, and cerebral cortex.

**Figure 6:**
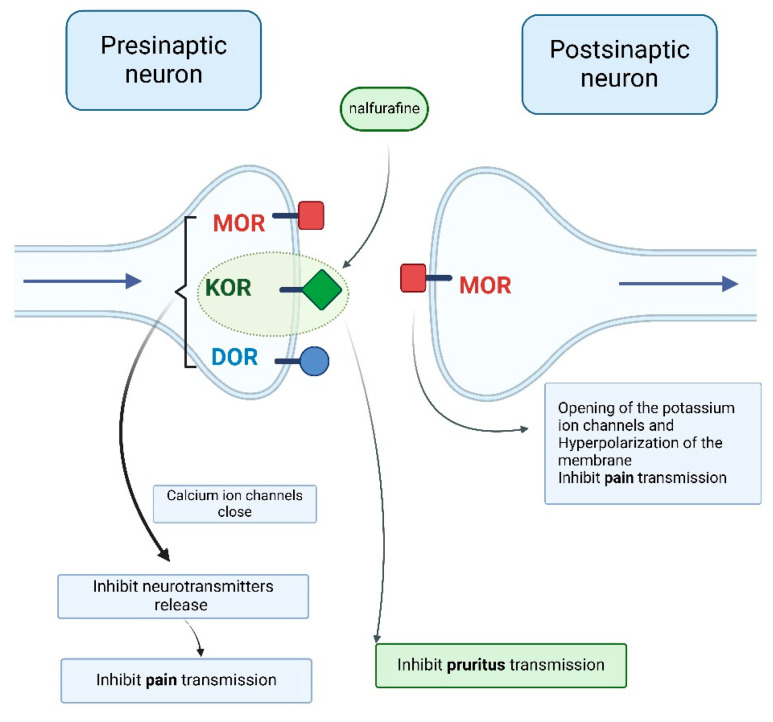
Opioid receptor stimulation producing different effects depending on the synaptic location [4,27] (Figure created in https://BioRender.com).

Opioid receptors are G-protein-coupled receptors that activate phospholipase C or inhibit adenylyl cyclase, producing different effects depending on their synaptic location. Presynaptically, opioid receptor activation closes calcium ion channels, inhibiting neurotransmitter release (e.g., acetylcholine, norepinephrine, serotonin, glutamate, and substance P). Postsynaptically, it opens potassium ion channels, leading to hyperpolarization and inhibitory potential (Figure 6) [26,27].

The endogenous peptides that activate opioid receptors include endorphins, enkephalins, and dynorphins, which accumulate in vesicles and are released into the synaptic cleft. These peptides modulate transmission of pain and pruritus in the brain, spinal cord, adrenal medulla, and neural plexus of the gut [3,27].

Opioid agonists mimic endogenous opioids by binding to these receptors. Strong agonists such as morphine, methadone, fentanyl, and heroin primarily act on MOR but can also affect KOR and DOR [3,26,27]. Their effects include acute pain relief and attenuation of pain’s emotional impact. Moderate agonists (e.g., codeine, hydrocodone, and oxycodone) provide mild-to-moderate analgesia and are often combined with anti-inflammatory drugs.

In addition to their analgesic effects, opioids can cause sedation, euphoria, respiratory depression, antitussive effects, gastrointestinal disturbances (nausea, vomiting, constipation), miosis, and tolerance or dependence [27]. Strong agonists like morphine and fentanyl are used to manage moderate-to-severe pain in cancer patients and during anesthesia. Morphine is also used for acute pulmonary edema due to its hemodynamic and calming effects. Codeine, a moderate opioid, is used in combination with acetaminophen and as a cough suppressant [27].

Opioid antagonists counteract opioid overdose by having a higher affinity for MOR than agonists. Examples include naloxone and naltrexone. Naloxone has a short duration (1–2 h), while naltrexone is long-acting (8–12 h) and is used to block the effects of herion and reduce cravings for ethanol.

A third class, agonist–antagonists, includes buprenorphine (a partial MOR agonist and KOR antagonist) and nalbuphine (a KOR agonist and MOR antagonist). These are primarily used for analgesia and to suppress alcohol withdrawal symptoms [27].

**Figure 7:**
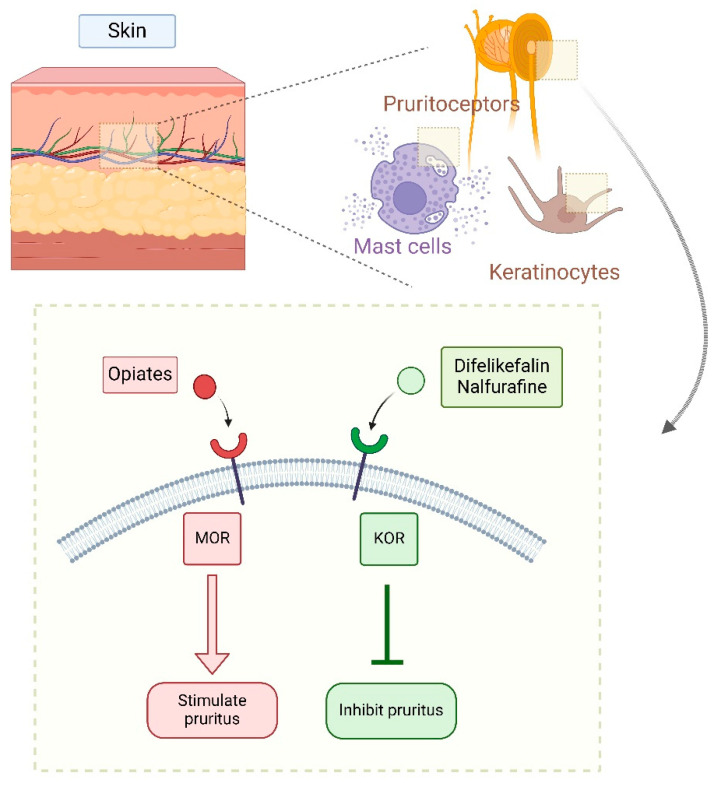
Peripheral effects of stimulation of specific opioid receptors [4,27] (Figure created in https://BioRender.com).

In the past two decades, two new KOR agonists have been developed for pruritus treatment in dialysis patients. Nalfurafine (central action) and difelikefalin (peripheral action) both inhibit pruritus pathways by stimulating KORs on cutaneous mast cells and keratinocytes (difelikefalin) or in the central nervous system, particularly the dorsal horn of the spinal cord (nalfurafine) [3,4] (Figure 6 and Figure 7).

Inflammation significantly impacts opioid-mediated pain and modulation of pruritus, as both share overlapping pathways. Opioid receptor density increases during inflammation, and certain cytokines (IL-6 and TNF-α) can have analgesic effects in inflamed tissues. Conversely, in noninflamed tissues, cytokines such as IL-1α, IL-1β, IL-6, and TNF-α contribute to hyperalgesia. The beneficial effects of opioid agonists and antagonists may stem from their ability to limit proinflammatory molecule release and reduce expression of adhesion molecules [4].

#### 7.1.1. Difelikefalin

Difelikefalin (DFK) acts as an agonist on the kappa-opioid receptor (KOR). Some alternative names for difelikefalin include 4-amino-1-(D-phenylalanyl-D-phenylalanyl-D-leucyl-D-lysyl) piperidine-4-carboxylic acid, KORSUVA, CKD-943, MR13A9, FE202845, and CR 845. Its structure consists of a small, hydrophilic peptide, which facilitates its distribution to peripheral nerves. Activation of peripheral opioid receptors on keratinocytes reduces the number of afferent pain signals transmitted to the central nervous system (CNS), which is generally responsible for itch mechanisms and nociception. Unlike other opioid drugs, DFK does not cross the blood-brain barrier, which is an important advantage as it does not cause CNS-related adverse effects such as respiratory depression. Moreover, being a selective KOR agonist provides an additional benefit, as it does not induce euphoria, a side effect commonly associated with mu-opioid receptor (MOR) stimulation [28].

DFK modulates pruritus pathways through various mechanisms [29,30]. It interrupts signal transmission along peripheral sensory C-fibers, particularly in the dorsal root ganglion. However, recent research suggests that DFK primarily acts on Aβ-fibers (large myelinated mechanoreceptors) rather than small unmyelinated pruriceptive C-fibers. The same study concluded that DFK may suppress itching independently of inflammatory mechanisms [29]. Nonetheless, because KOR is widely distributed in the periphery and has been associated with anti-inflammatory effects—such as inhibition of neurogenic inflammation and nociceptor sensitization—DFK’s complete independence from inflammatory pathways remains uncertain (Figure 7).

**Figure 8:**
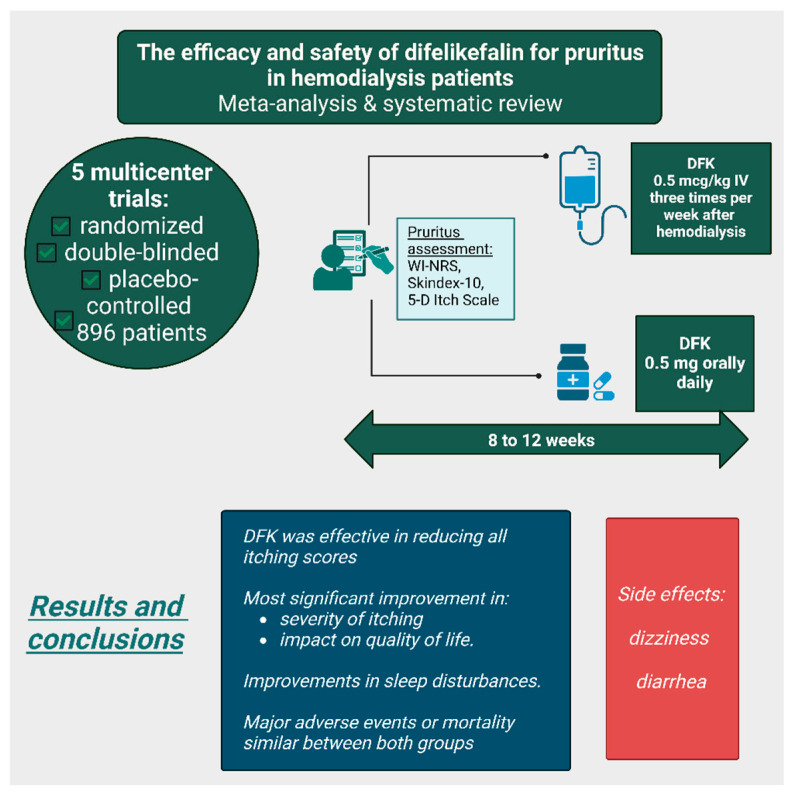
The efficacy and safety of difelikefalin treatment for uremic pruritus according to a meta-analysis and systematic review [31]. (Figure created in https://BioRender.com).

A meta-analysis and systematic review examined five multicenter, randomized, double-blinded, placebo-controlled trials, including a total of 896 patients; two of these studies were conducted in Japan and three in the United States (Figure 8). In these trials, DFK was administered either intravenously (0.5 mcg/kg IV three times per week after hemodialysis) or orally (0.5 mg daily) for 8 to 12 weeks. The impact of pruritus was assessed before, during, and after therapy using various itch scales, including the WI-NRS, Skindex-10, and 5-D itch scale. DFK was effective in reducing all itching scores, with the most significant improvement observed in the Skindex-10 total score, which evaluates the severity of itching, as well as its impact on mental and physical quality of life. Additionally, patients reported improvements in sleep disturbances. However, dizziness and diarrhea were more frequently observed in the DFK group compared with the placebo group. Nevertheless, the rates of major adverse events and mortality were similar between both groups. The meta-analysis concluded that, based on available data, DFK is a promising therapy for CKD-associated pruritus, though further robust studies are necessary to confirm its long-term benefits [31].

The most frequently reported side effects in phase 3 clinical trials among patients who received a twofold dose of DFK were diarrhea, vomiting, and dizziness. At a tenfold dose increase, 20% to 60% of participants experienced paresthesia, hypoesthesia, and somnolence; however, these effects lasted no longer than 90 min and resolved spontaneously without the need for medical intervention. That study also evaluated DFK’s potential to cause respiratory depression in healthy subjects, reporting a lowest observed respiratory rate of 14 breaths per minute—above the study’s predefined threshold of 10 breaths per minute for respiratory depression [28].

#### 7.1.2. Nalfurafine

**Figure 9:**
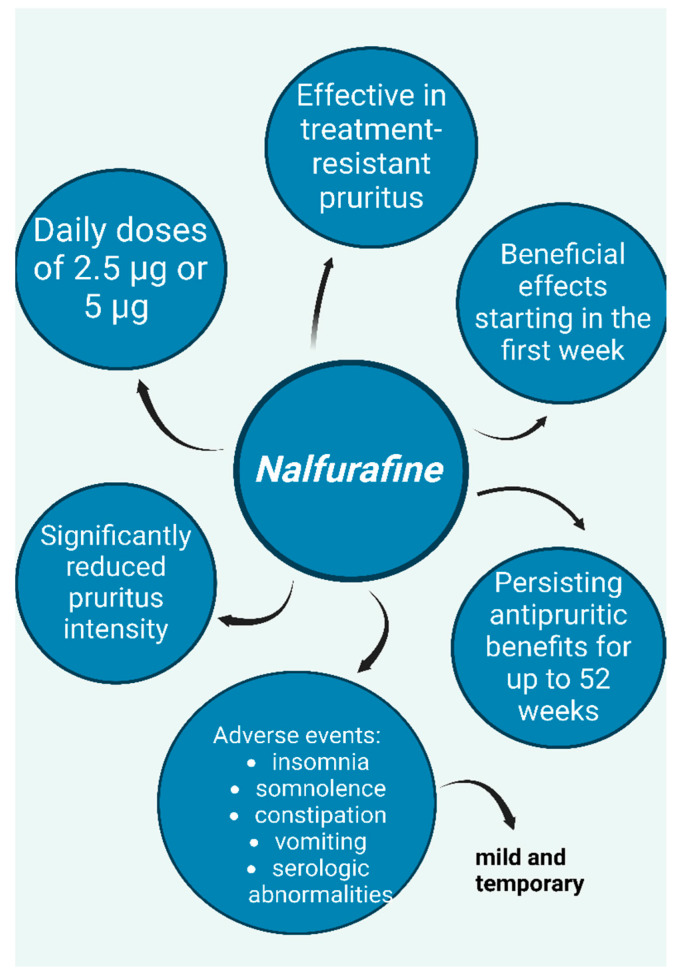
Nalfurafine, a kappa-opioid receptor (KOR) agonist—potential option for treatment of resistant uremic pruritus; graphic depicts positive effects, adverse events, and dosing according to studies [32,33,34,35,36]. (Figure created in https://BioRender.com).

A recent meta-analysis reviewed multiple Japanese clinical trials on the clinical efficacy and safety of nalfurafine, a kappa-opioid receptor (KOR) agonist with central action, in the treatment of pruritus from various causes, particularly uremic pruritus in dialysis patients and hepatic pruritus in individuals with chronic liver disease [32], (Figure 9).

Regarding uremic pruritus in hemodialysis patients, a prospective, double-blind, placebo-controlled study conducted in Japan found that nalfurafine significantly reduced treatment-resistant pruritus at daily doses of 5 μg and 2.5 μg, with positive effects appearing within the first week of treatment [32,33]. Furthermore, a long-term study demonstrated that the antipruritic effects of nalfurafine persisted for up to 52 weeks [32,34]. The safety profile of nalfurafine was evaluated in both studies, with reported adverse effects including insomnia, somnolence, constipation, and transient serologic abnormalities in some hormones. However, these side effects were mild and temporary [33,34]. Additionally, both studies employed questionnaires to assess the potential for drug dependence and concluded that nalfurafine has a low risk of abuse [33,34].

Similar results were observed in studies investigating resistant pruritus in peritoneal dialysis patients. An open-label study reported significant symptom improvement and enhanced quality of life after progressively increasing doses of nalfurafine over four weeks (2.5 μg daily during weeks 1 and 2; 5 μg daily during weeks 3 and 4) [32,35]. The reported adverse effects—somnolence, hormone-related blood abnormalities, and vomiting—were consistent with those observed in previous studies and resolved spontaneously without requiring specific treatment, further supporting the safety of the therapy.

A recent phase 3 multicenter bridging study conducted in China, including 141 hemodialysis patients with refractory pruritus, reached similar conclusions [36]. Patients were administered nalfurafine at daily doses of 5 μg or 2.5 μg, or a placebo, for 14 days, with the severity of their pruritus assessed using the visual analog scale (VAS). A daily dose of 5 μg was effective in alleviating pruritus, whereas the 2.5 μg dose also reduced itching symptoms, but the VAS difference was not statistically significant. Adverse drug reactions were reported in 49.1% of patients in the 5 μg group, 38.6% in the 2.5 μg group, and 33.3% in the placebo group, with insomnia being the most frequently encountered side effect [36].

### 7.2. Gabapentinoids

Research suggests that gabapentinoids demonstrate promising efficacy in the treatment of uremic pruritus and are among the most extensively investigated pharmacological classes for this condition.

A randomized, double-blind study conducted in the Philippines evaluated the efficacy of topical gabapentin cream for relief of uremic pruritus. The study included 30 patients undergoing hemodialysis for at least eight weeks with a baseline VAS score of ≥5 for pruritus. It found that topical 6% gabapentin cream, applied for two weeks, resulted in a mean VAS score reduction of −4.6, indicating significantly improved pruritus compared with the placebo (permeation cream) [37].

**Figure 10:**
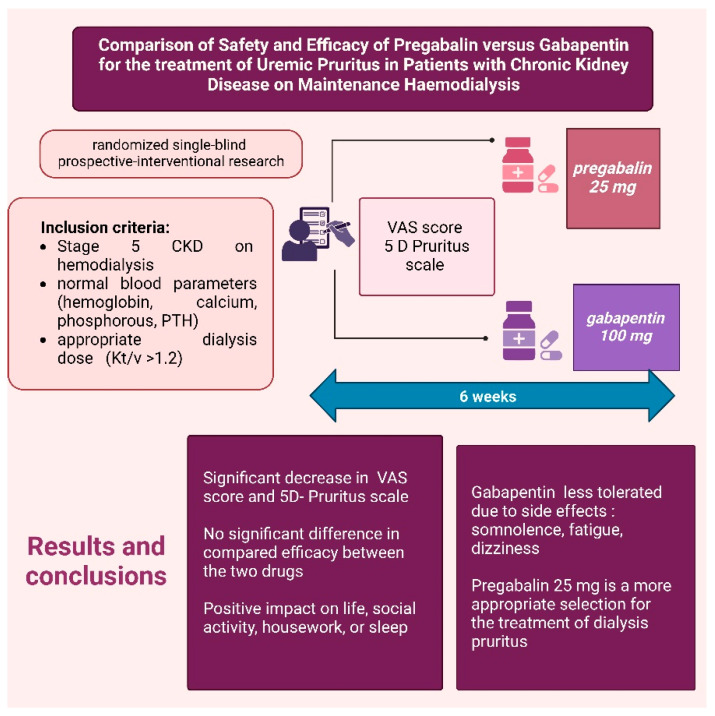
Efficacy of Pregabalin vs. Gabapentin in Alleviating Pruritus [39]. (Figure created in https://BioRender.com).

Numerous clinical studies have aimed to compare the efficacy of gabapentinoids, particularly gabapentin and pregabalin. A randomized, single-blind, prospective interventional study investigated the efficacy and safety of gabapentin and pregabalin in hemodialysis patients experiencing severe pruritus (Figure 10). The study included patients with Stage 5 CKD on hemodialysis, with normal blood parameters (hemoglobin, calcium, phosphorus, and parathyroid hormone) and an adequate dialysis dose (Kt/V > 1.2). Participants received either pregabalin 25 mg or gabapentin 100 mg for six weeks, with VAS scores and the 5-D Itch Scale assessed before and after treatment. The results demonstrated significant reductions in both scales for both drugs, with no statistically significant difference in efficacy. Both pregabalin and gabapentin notably reduced the duration and intensity of pruritus, as well as improving quality-of-life factors such as social activity, household tasks, and sleep. However, gabapentin was less well tolerated in the study population due to side effects such as somnolence, fatigue, and dizziness. Therefore, the authors concluded that pregabalin 25 mg may be a more appropriate option for the treatment of dialysis-associated pruritus [38].

Researchers have also compared the efficacy of gabapentinoids with other pharmacological classes, such as pregabalin versus sertraline, in managing uremic pruritus. A randomized trial involving 62 patients with uremic pruritus assessed the effects of pregabalin and sertraline over four weeks. The inclusion criteria required patients with CKD undergoing hemodialysis twice weekly who had pruritus that was unresponsive to antihistamines, anti-inflammatory agents, or topical treatments. The pregabalin group received 25 mg for the first week, increasing progressively to 50 mg in the second week and 75 mg in the third and fourth weeks. The sertraline group started with 25 mg for the first week, increasing to 50 mg for weeks 2, 3, and 4. The lowest effective dose was used if improvements in symptoms were observed. The study was conducted over four weeks, with 5-D itch scale assessments every two weeks. Results demonstrated that both treatments effectively reduced pruritus intensity, with no significant difference in 5-D itch scale scores between the two groups. However, the authors noted that the study was not designed to assess the side effects of the tested drugs, representing a limitation of the research [39].

### 7.3. Antidepressants

While the time required for sertraline to take effect means it is used less, and though it is not always considered practical, sertraline may still be a viable option for patients with comorbid depression [2].

A double-blind, placebo-controlled, multicenter study conducted in Egypt investigated the efficacy of sertraline in managing uremic pruritus [14]. The study enrolled 60 long-term hemodialysis patients experiencing mild, moderate, or severe pruritus. Participants were randomly assigned to receive either sertraline (50 mg twice daily, totaling 100 mg/day) or a placebo for 8 weeks. Effectiveness was assessed using the VAS and the 5-D itch scale at baseline and every two weeks. The results demonstrated a significant improvement in pruritus with sertraline treatment. Patients receiving sertraline showed a statistically significant reduction in both VAS and 5-D itch scale scores, whereas the placebo group showed no meaningful improvement and in some cases, worsening of symptoms. Among patients with severe pruritus (approximately one-third of participants), sertraline markedly reduced VAS scores (from 33.3% to 6.9%) and 5-D itch scale scores (from 36.7% to 17.2%). Interestingly, the study found a positive correlation between the severity of pruritus (as measured by both scales) and serum levels of urea and ferritin, but no correlation with calcium, phosphorus, parathyroid hormone (PTH), serum albumin, or Kt/V. The authors attributed these findings to the characteristics of the study population.

## 8. Global Analysis of Treatment Options Regarding Efficacy and Adverse Reactions in Pruritus Associated with Chronic Kidney Disease

A 2024 updated meta-analysis including 91 studies and approximately 4650 participants evaluated pharmacological interventions for itching in palliative care settings [40] In patients with uremic pruritus, gabapentinoid therapy resulted in a significant reduction in symptoms, supported by a moderate level of evidence certainty. In contrast, kappa-opioid receptor agonists (difelikefalin, nalbuphine, and nalfurafine) led to only modest improvement, albeit with a high level of evidence certainty. The investigators concluded that GABA analogues were more effective than kappa-opioid agonists. However, the evidence supporting other therapeutic options remains limited, with low to very low levels of certainty.

For example, fish oil/omega-3 fatty acids and topical capsaicin were associated with reduced pruritus, though the confidence in these findings was constrained by low certainty. Cromolyn sodium showed the lowest level of certainty for itch relief. Additionally, treatments such as ondansetron, zinc sulfate, and several others were deemed entirely ineffective. A 2024 Chinese meta-analysis evaluated the efficacy and safety of various drugs for uremic pruritus in 22 randomized controlled trials (RCTs) involving 2877 hemodialysis patients [41]. The treatments assessed included the following:

- Gabapentinoids: gabapentin (137 patients), pregabalin (21);
- Kappa-opioid receptor agonists: difelikefalin (763), nalfurafine (394), nalbuphine (248);
- Other agents: nemolizumab (41), montelukast (40), sertraline (30), dexchlorpheniramine (30), ketotifen (26), cromolyn sodium (21);
- Placebo: 1049 patients;
- Additional therapies: thalidomide, melatonin, nicotinamide, hydroxyzine.

Regarding pruritus relief, the most effective treatments, in order, were gabapentin, cromolyn sodium, hydroxyzine, montelukast, melatonin, sertraline, nalfurafine, nalbuphine, difelikefalin, and nicotinamide. The medications with the highest patient-reported response rates were thalidomide, gabapentin, nalfurafine, and difelikefalin. The risk of adverse reactions was highest with gabapentin, followed by ketotifen, dexchlorpheniramine, difelikefalin, nalfurafine, pregabalin, nemolizumab, and cromolyn sodium. Seven studies assessed nausea in CKD-aP patients undergoing antipruritic therapy [41]; cromolyn sodium was associated with less nausea than even the placebo, while the highest rates were seen with nalbuphine, followed by difelikefalin, nalfurafine, and sertraline. Six studies reported that diarrhea was least likely with cromolyn sodium and difelikefalin, but most common with sertraline, followed by nemolizumab, nalfurafine, and difelikefalin. Somnolence was least associated with difelikefalin and ketotifen, and most associated with nalbuphine and nalfurafine. Dizziness was most frequent in patients receiving gabapentin, and least common in those treated with difelikefalin or ketotifen.

## 9. Conclusions

At present, no therapeutic intervention has demonstrated clearly superior efficacy, making it difficult to establish a definitive treatment trend. Consequently, there is a pressing need for well-designed comparative clinical trials encompassing a broad spectrum of therapeutic options. These studies should ideally include large patient cohorts and be conducted over extended periods to ensure the reliability and generalizability of their results.

In our opinion, further research should directly compare efficacy and safety between two or more therapeutic options, especially those that are newly discovered and show promise (difelikefalin, nalfurafine, gabapentinoids), without neglecting classical therapies that are relatively easy to administer (emollients, UVB therapy).

Managing such a complex condition with multiple therapeutic options can be challenging and frustrating for both patients and physicians. The key is to avoid relying on a single therapy and instead tailor the treatment approach to each patient’s individual response. While some patients may respond well to a single treatment, others may require a combination of the therapies mentioned above.

What truly makes a difference is an ongoing collaboration between physician and patient, taking into account patient preferences, closely monitoring symptom changes, and regularly seeking feedback after each treatment adjustment.

## Figures and Tables

**Table 1:** Main pathologic mechanisms in uremic pruritus and their corresponding treatment options.

Treatment	Pathologic Mechanism
Emollients rich in glycerol and paraffin	Skin barrier dysfunction and xerosis
SPF creams	Skin barrier dysfunction and xerosis
Narrowband ultraviolet B phototherapy	Increased pro-inflammatory cytokines, immune system abnormality
Nemolizumab	Increased pro-inflammatory cytokines, immune system abnormality
Nalfurafine	Opioid system dysregulation
Difelikefalin	Opioid system dysregulation
Pregabalin	Similar pain neurotransmitters and pathways
Gabapentin	Similar pain neurotransmitters and pathways
Sertraline	Depression comorbidity

## Data Availability

This review summarizes data reported in the literature and it does not report primary data.
